# Lead Immobilization in Soil and Uptake Reduction in *Brassica chinensis* Using Sepiolite-Supported Manganese Ferrite

**DOI:** 10.3390/plants14193077

**Published:** 2025-10-05

**Authors:** Fengzhuo Geng, Yaping Lyu, Liansheng Ma, Yin Zhou, Jiayue Shi, Roland Bol, Peng Zhang, Iseult Lynch, Xiuli Dang

**Affiliations:** 1 College of Land and Environment, National Engineering Research Center for Efficient Utilization of Soil and Fertilizer Resources, Key Laboratory of Arable Land Conservation in Northeast China, Ministry of Agriculture and Rural Affairs, Shenyang Agricultural University, Shenyang 110866, China; 2024220452@stu.syau.edu.cn (F.G.); ss15032928639@163.com (L.M.); zhouy20252025@163.com (Y.Z.); Sjy020517@163.com (J.S.); 2 Department of Environmental Science and Engineering, University of Science and Technology of China, Hefei 230026, China; 13001012362@163.com (Y.L.); zhangpeng1987@ustc.edu.cn (P.Z.); 3 Institute of Bio- and Geosciences, Agrosphere (IBG-3), Forschungszentrum Jülich GmbH, 52425 Jülich, Germany; r.bol@fz-juelich.de; 4 School of Geography, Earth and Environmental Sciences, University of Birmingham, Edgbaston, Birmingham B15 2TT, UK; i.lynch@bham.ac.uk

**Keywords:** manganese ferrite nanoparticles (MnFe_2_O_4_ NPs), adsorption, soil remediation, Pak Choi, nano-composite, lead toxicity

## Abstract

Lead (Pb) in soil poses serious environmental and health risks, and its removal requires complex and costly treatment methods to meet strict regulatory standards. To effectively address this challenge, innovative and efficient techniques are essential. Sepiolite-supported MnFe_2_O_4_ (MnFe_2_O_4_/SEP) composites were synthesized via a chemical co-precipitation method. The effects of MnFe_2_O_4_/SEP on soil pH, cation exchange capacity (CEC), available Pb content, Pb^2+^ uptake, and the activities of antioxidant enzymes in *Brassica chinensis* (Pak Choi) were examined. MnFe_2_O_4_/SEP showed superior Pb^2+^ adsorption compared to SEP alone, fitting Langmuir models, Dubinin-Radushkevich (D-R) models, Temkin models and pseudo-second-order kinetics. The maximum adsorption capacities at 298, 308, and 318 K were 459, 500 and 549 mg·g^−1^, respectively. XPS analysis indicated that chemisorption achieved through ion exchange between Pb^2+^ and H^+^ was the main mechanism. MnFe_2_O_4_/SEP increased the soil pH by 0.2–1.5 units and CEC by 18–47%, while reducing available Pb by 12–83%. After treatment with MnFe_2_O_4_/SEP, acid-extractable and reducible Pb in the soil decreased by 14% and 39%, while oxidizable and residual Pb increased by 26% and 21%, respectively. In *Brassica chinensis*, MnFe_2_O_4_/SEP reduced Pb^2+^ uptake by 76%, increased chlorophyll content by 36%, and decreased malondialdehyde (MDA) levels by 36%. The activities of antioxidant enzymes—superoxide dismutase (SOD), peroxidase (POD), and catalase (CAT)—were decreased by 29%, 38% and 17%, respectively. These findings demonstrate that MnFe_2_O_4_/SEP is an efficient Pb^2+^ adsorbent that immobilizes Pb in soil mainly through ion exchange, thereby providing a highly effective strategy for remediating Pb-contaminated soils and improving plant health.

## 1. Introduction

In recent decades, rapid economic development has accelerated the release of heavy metals, particularly lead (Pb), into the environment. It is estimated that approximately 783,000 tons of Pb have been discharged globally, with soils acting as the ultimate sink and consequently experiencing significant contamination [1]. A national survey of soil pollution in China conducted from 2005 to 2013 showed that inorganic pollution accounted for the largest proportion (16.1%) of environmental contamination among all types. Furthermore, according to the latest survey by the Ministry of Ecology and Environment (2023–2024), 7.2% of China’s farmland soil exceeds the Pb^2+^ limits set by national standards. Vegetables play an important role in the Chinese diet, and Pak Choi (*Brassica chinensis*), a common leafy green, is widely consumed. However, Pb^2+^ is readily absorbed by *Brassica chinensis* from contaminated soil and transferred to humans through the food chain, posing serious health risks [2,3]. Notably, Pb^2+^ exposure can severely impair the nervous system and vital organs, with children being particularly vulnerable to its detrimental effects on growth and development [4]. Therefore, developing a simple and low-cost method to remove Pb^2+^ from soil has become a critical issue. Chemical precipitation, electrochemical methods, ion exchange and adsorption methods are the popular technologies for remediation of Pb-contaminated farmland [5]. Among these methods, adsorption is the preferred approach for remediating Pb-contaminated farmland due to its high efficiency and low cost [6]. Hence, the development of highly effective adsorbents for heavy metal removal is a significant trend in current environmental research, with a variety of materials such as carbon-based compounds, nanoparticles, and clay minerals being investigated for their ability to remove heavy metals from wastewater and soil [7,8,9].

Manganese ferrite nanoparticles (MnFe_2_O_4_ NPs) have gained prominence due to their remarkable physicochemical, optical, and magnetic features, including low magnetic loss, high permeability, and robust chemical stability. Un-agglomerated MnFe_2_O_4_ has a large specific surface area due to its nanoscale, and a large number of hydroxyl groups on the surface make it a potential substrate for Pb^2+^ adsorption [10]. However, the magnetic nature of MnFe_2_O_4_ NPs often leads to their agglomeration, which makes the actual surface area for adsorption, and the absorption efficiency much lower than the theoretical value [11]. This issue is typically addressed by using carbon-based or silica-based materials as stabilizing supports for MnFe_2_O_4_ NPs to reduce their agglomeration potential [12]. Loading magnetic MnFe_2_O_4_ onto a sludge biochar composite has been demonstrated to effectively prevent the agglomeration of MnFe_2_O_4_ and improve the adsorption efficiency of Pb^2+^ [13].

Natural clay minerals are aluminosilicates with a layered structure. Common clay minerals include zeolite, sepiolite (SEP) and montmorillonite, which are natural materials with adsorption properties for Pb^2+^ [14]. SEP, in particular, stands out as a fibrous sheet-structured silicate clay mineral, rich in silicon and magnesium. The lamellar fiber structure of SEP has a large specific surface area, which is not only an excellent natural material for adsorption of Pb^2+^, but also an ideal carrier for nanomaterials. Fu et al. (2015) successfully prepared SEP-supported nano-zero-valent iron for efficient removal of Cr^6+^ and Pb^2+^ from groundwater [15]. The results showed that the agglomeration phenomenon of nano-zero-valent iron after loading was reduced, and the removal rate of Cr^6+^ and Pb^2+^ in water was increased. Therefore, SEP is a potential support for MnFe_2_O_4_ NPs. The synergy between MnFe_2_O_4_ and SEP in a composite form is anticipated to not only preserve but also amplify the inherent adsorption qualities of each individual component, harnessing the best of both worlds for improved efficacy in heavy metal removal.

In this study, a sepiolite-supported manganese ferrite nanoparticles (MnFe_2_O_4_/SEP) composite magnetic material was prepared and characterized by scanning electron microscope with energy dispersive spectroscopy (SEM–EDS), X-ray diffraction (XRD), Fourier Transform infrared (FT-IR) spectroscopy and X-ray Photoelectron Spectroscopy (XPS). The adsorption behavior and properties of Pb^2+^ in water were studied under the conditions of different material dosage, pH, adsorption temperature and adsorption time. In addition, through adsorption kinetics, adsorption thermodynamics calculations, and isothermal adsorption fitting, combined with XPS characterization, the adsorption mechanism for Pb^2+^ was explored. Finally, the remediation effects of MnFe_2_O_4_/SEP in soil were evaluated by measuring the Pb availability, Pb^2+^ content, enzyme activities and the antioxidative responses in the plant *Brassica chinensis* grown in the Pb-contaminated soil.

## 2. Results and Discussion

### 2.1. Adsorbent Characterization

The morphologies and elemental compositions of raw SEP and MnFe_2_O_4_/SEP are shown in Figure S1. SEP exhibited a typical fibrous lamellar structure with a smooth surface (Figure S1a) and was mainly composed of Ca, Mg, O, and Si (Figure S1c). After modification, the surface became rough with granular deposits, while MnFe_2_O_4_ aggregation was effectively prevented (Figure S1b). Energy spectrum analysis confirmed the presence of Mn and Fe in the composite (Figure S1d and Table S2). The content ratio of the two elements was 1:2, and compared with SEP, their contents increased by 11% and 22% respectively, indicating successful MnFe_2_O_4_ loading. The specific surface area increased from 34.04 to 112.61 m^2^·g^−1^ (Table S1), providing 3.3 times more binding sites for heavy metals. These improvements suggest that MnFe_2_O_4_/SEP has strong potential as an efficient Pb^2+^ adsorbent. XRD analysis confirmed the crystal structures of SEP, MnFe_2_O_4_, and MnFe_2_O_4_/SEP (Figure S1e). Reflections at 7.25°, 27.91°, 39.86°, and 43.64° corresponded to SEP (JCPDS 26-1226), while additional peaks at 34.88°, 36.47°, 52.59°, and 61.51° (JCPDS 38-0430) verified the presence of MnFe_2_O_4_. FT-IR spectra (Figure S1f) further confirmed MnFe_2_O_4_ loading, with Mn–O (680 cm^−1^) and Fe–O (484 cm^−1^) vibrations observed. The decreased –OH signal at 3673 cm^−1^ and the broadened band at 3000–3450 cm^−1^ suggested loss of bonded water and formation of new functional groups [16], which are expected to enhance the cation exchange capacity and Pb^2+^ adsorption [17].

### 2.2. Adsorption Properties of MnFe_2_O_4_/SEP

**Figure 1:**
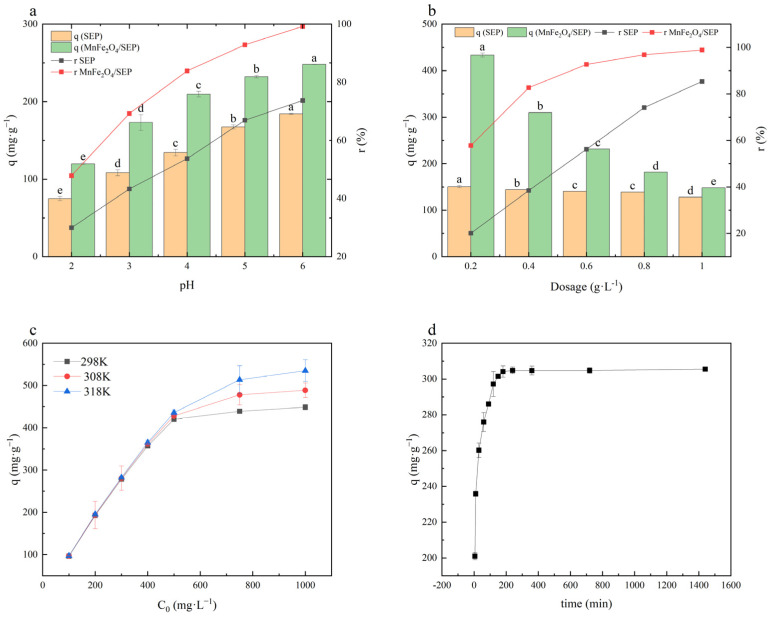
Effect of pH (**a**), dosage (**b**), initial concentration and temperature (**c**) and time (**d**) on the Pb^2+^ adsorption quantity (q) and removal rate (r) by SEP and MnFe_2_O_4_/SEP. Data are presented as mean ± SE (*n* = 3). Different lowercase letters indicate significant difference between different treatments (*p* < 0.05).

The adsorption behavior of Pb^2+^ by SEP and MnFe_2_O_4_/SEP was strongly influenced by pH (Figure 1a). With increasing pH, both adsorption capacity and removal efficiency increased due to the reduced competition between H^+^ and Pb^2+^ for active sites [18]. Maximum adsorption was achieved at pH 6, where MnFe_2_O_4_/SEP reached nearly 100% removal, significantly outperforming SEP, indicating more available binding sites in the composite. Adsorbent dosage also affected Pb^2+^ removal (Figure 1b). At lower dosages, adsorption approached maximum capacity, whereas higher dosages mainly improved removal efficiency [19]. An optimal dosage of 0.6 g·L^−1^ MnFe_2_O_4_/SEP achieved 92.68% Pb^2+^ removal, outperforming bare SEP. Isothermal adsorption results further demonstrated that Pb^2+^ uptake by MnFe_2_O_4_/SEP increased with initial concentration and temperature, reaching equilibrium above 500 mg·L^−1^ (Figure 1c). Langmuir isotherm fitting provided the best description of the process, with theoretical maximum adsorption capacities of 458, 500, and 549 mg·g^−1^ at 298, 308, and 318 K, respectively (Table 1), confirming monolayer chemisorption as the dominant mechanism. D-R model fitting yielded characteristic adsorption energies (E) of 0.37, 0.43 and 0.49 kJ·mol^−1^ at 298, 308, and 318 K (Table 1). Since all values were below 8 kJ·mol^−1^, the adsorption process can be attributed to physical adsorption [20]. In contrast, the Temkin model fitting produced adsorption heat constants (b) of 28.01, 30.06, and 31.80 kJ·mol^−1^ at 298, 308, and 318 K, respectively. These values fall within the range of 20–40 kJ·mol^−1^, suggesting the involvement of chemical adsorption [21]. Taken together, these findings indicate that the adsorption of Pb^2+^ by MnFe_2_O_4_/SEP is governed by the combined contributions of both physical and chemical adsorption.

### 2.3. Adsorption Kinetics

The kinetic effect of Pb^2+^ adsorption by the adsorbent was investigated through studies on the adsorption process at different time intervals. As shown in Figure 1d, the Pb^2+^ adsorption capacity of MnFe_2_O_4_/SEP increased rapidly within 1 h, slowed between 1–3 h, and reached equilibrium after 3 h. Initially, favorable conditions—including abundant adsorption sites on MnFe_2_O_4_/SEP and high Pb^2+^ concentration—caused rapid adsorption within the first hour. With time extension (2–3 h), reduced surface sites decreased pore diffusion rates, but electrostatic repulsion existed between the unadsorbed Pb^2+^ in the solution and the Pb^2+^ already bound to the surface of MnFe_2_O_4_/SEP [22,23] until equilibrium was achieved with complete site occupation. Kinetic fitting results (Figure S2, Table S3) showed the pseudo-second-order model best described the adsorption process. This model, which encompasses both internal diffusion and surface adsorption of the composite, indicates that the Pb^2+^ adsorption mechanism of MnFe_2_O_4_/SEP results from multiple combined effects [24].

### 2.4. Thermodynamic Study

**Figure 2:**
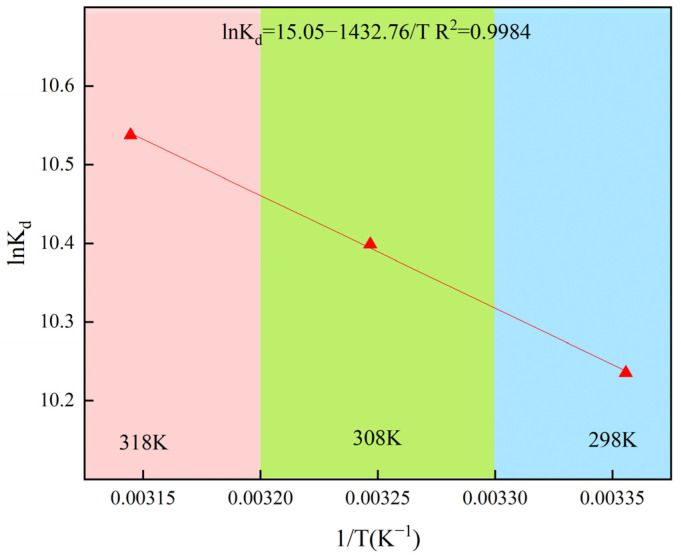
Van’t Hoff plot for the adsorption of Pb^2+^ onto MnFe_2_O_4_/SEP as a function of temperature.

By analyzing the adsorption process of Pb^2+^ by MnFe_2_O_4_/SEP at 298, 308 and 318 K, the thermodynamic behavior of adsorption was further determined. The thermodynamic fitting results are shown in Figure 2. The results showed that the process for MnFe_2_O_4_/SEP adsorption of Pb^2+^ was endothermic (ΔH was 11.91 kJ·mol^−1^) and that increasing the temperature increased the disorder of the adsorption system and thus increased the amount of Pb^2+^ adsorbed by MnFe_2_O_4_/SEP. The adsorption process was spontaneous, as indicated by the negative ΔG values at all three temperatures (−25.38, −26.63, and −27.88 kJ·mol^−1^, respectively). Moreover, the ΔG values were all below 50 kJ·mol^−1^, suggesting that the removal of Pb^2+^ by MnFe_2_O_4_/SEP was primarily controlled by chemical interactions [25]. Previously, Langmuir fitting indicated that adsorption mainly occurs on the material surface. The characteristic adsorption energy E from D-R fitting (E < 8 kJ·mol^−1^) confirms physical interactions, while the adsorption heat constant b from Temkin fitting (20–40 kJ·mol^−1^) suggests chemical interactions—together proving a physico-chemical synergistic adsorption process. Pseudo-first-order kinetics shows that Pb^2+^ physically diffuses to the material surface in the initial adsorption stage, and pseudo-second-order kinetics indicates chemical adsorption dominates the process. Based on the above comprehensive analysis, the adsorption mechanism of MnFe_2_O_4_/SEP can be summarized as a spontaneous process dominated by chemical adsorption with physical adsorption as a supplement, under the mode of monolayer surface adsorption.

### 2.5. Reusability Assessment

**Figure 3:**
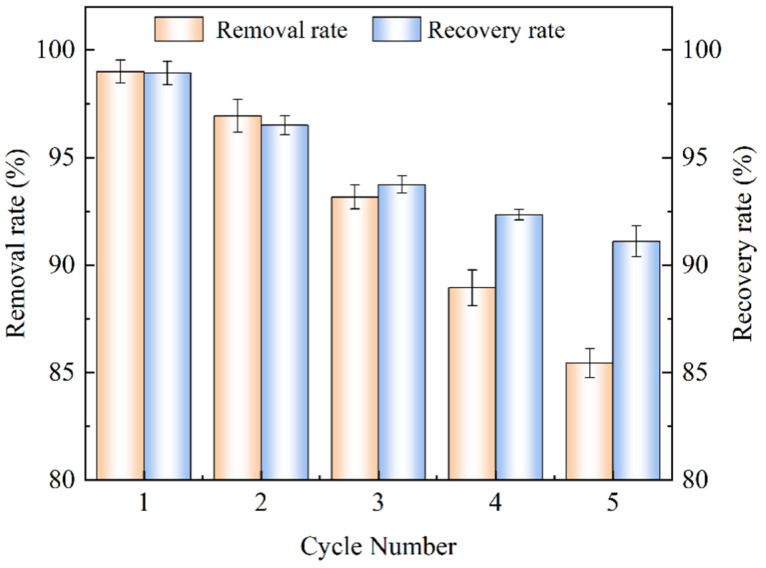
Removal rate of Pb^2+^ and recovery rate of MnFe_2_O_4_/SEP after five adsorption–desorption cycles using MnFe_2_O_4_/SEP. Data are presented as mean ± SD with *n* = 3 independent replicates.

The regeneration ability of MnFe_2_O_4_/SEP was evaluated by repeating the adsorption–desorption cycle five times. As shown in Figure 3, the Pb^2+^ removal rate and the MnFe_2_O_4_/SEP recovery rate gradually decreased with an increasing number of adsorption–desorption cycles. There are two possible reasons for this; one is the loss of adsorbent in the process of the adsorption and desorption cycle, and the other is that a small number of adsorption sites may be lost in the process of desorption, resulting in the reduction of removal rate over time. During the five cycles, the gap between the removal rate of Pb^2+^ and the recovery rate of the material became larger and larger, and the main reason for the decrease of Pb^2+^ removal rate was the inactivation of adsorption sites on the surface of MnFe_2_O_4_/SEP. However, after the fifth cycle, the removal rate still reached 85.4%. Therefore, MnFe_2_O_4_/SEP has a certain inherent stability and is expected to become a new kind of soil treatment material which is easy to prepare and economical.

### 2.6. Adsorption Mechanism

**Figure 4:**
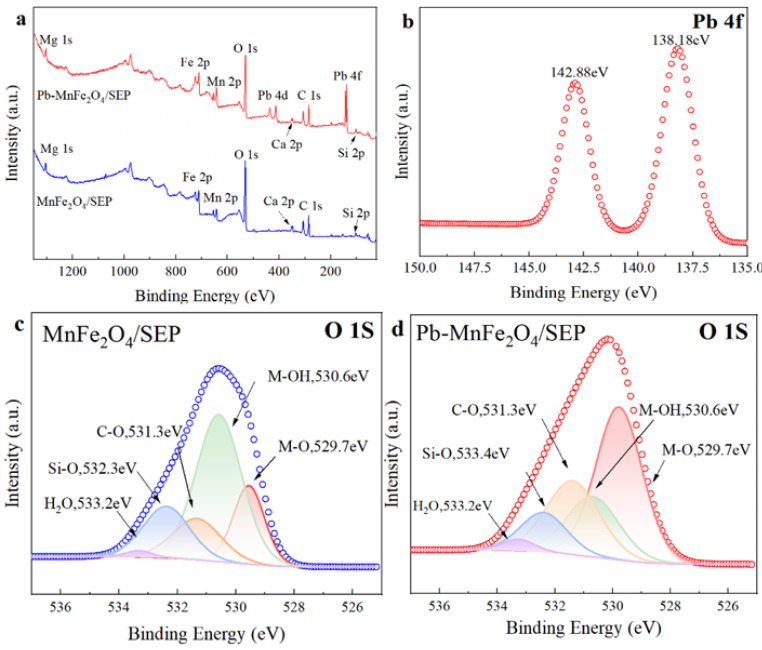
Characterization of the binding mechanisms, (**a**) XPS survey spectra of MnFe_2_O_4_/SEP before and after Pd^2+^ adsorption, (**b**) high-resolution XPS spectra of Pb4f, (**c**,**d**) fitting results of O1s before and after Pb^2+^ adsorption.

The XPS full spectrum scanning results are shown in Figure 4a. In addition to Mg, Ca, Fe, Mn, O, Si, and other elements contained in the material itself, the adsorbed material showed the characteristic absorption peaks of Pb 4d and Pb 4f, indicating that Pb^2+^ has been successfully bound to the surface of MnFe_2_O_4_/SEP. Figure 4b shows the high-resolution energy spectrum of Pb 4f, revealing that the shift of characteristic peaks relative to the standard binding energy is consistent with the characteristic adsorption energy E (average value of 0.4 kJ·mol^−1^) calculated by the D-R isotherm model and the adsorption heat constant b (average value of 30 kJ·mol^−1^) from the Temkin model. This consistency confirms that both physical and chemical interactions coexist in the adsorption process. The characteristic spectra of the O 1s orbitals of MnFe_2_O_4_/SEP and Pb-MnFe_2_O_4_/SEP are shown in Figure 4c,d. The characteristic peaks of O 1s can be fitted to five forms of O [26]. The peak area of metal hydroxide decreased significantly after adsorption, and the peak area of metal oxide increased significantly which may be caused by Pb^2+^ replacing H^+^ at the adsorption site. Before and after adsorption, the binding energy of Si-O bonds shifted. This shift may be attributed to the replacement of H in Si-OH groups by part of Pb, which forms Si-O-Pb bonds. The formation of Si-O-Pb bonds causes the binding energy of Si-O bonds to shift, thereby enabling Pb to be complexed on the surface and within the pores of the composite material. This is consistent with the indication of the pseudo-second-order kinetic model that chemical adsorption is the rate-limiting step, and it is consistent with the characteristics of monolayer adsorption on uniform active sites revealed by the Langmuir model, suggesting that the chemical complexation between Pb^2+^ and active sites constitutes the core mechanism of the adsorption process. In addition, the pseudo-first-order kinetic model reveals the existence of a physical diffusion process in the initial stage of adsorption, which is corroborated by the physical interaction reflected by the D-R model. This indicates that Pb^2+^ first diffuses to the material surface through physical attraction, laying the foundation for subsequent chemical adsorption.

In summary, the adsorption mechanism of Pb^2+^ from aqueous solutions by the MnFe_2_O_4_/SEP composite mainly involves physical adsorption and chemical adsorption, with chemical adsorption being dominant. Ion exchange is primarily manifested in the complexation of Pb^2+^ on the composite surface by the M-OH and Si-OH groups on the composite surface.

Si-OH + Pb^2+^ = [(Si-O)Pb]^+^ + H^+^ (1)

2Si-OH + Pb^2+^ = (Si-O)_2_Pb + 2H^+^ (2)

M-OH + Pb^2+^ = [(M-O)Pb]^+^ + H^+^ (3)

2M-OH + Pb^2+^ = (M-O)_2_Pb + 2H^+^ (4)

### 2.7. Application of MnFe_2_O_4_/SEP in Pb-Contaminated Soil

#### 2.7.1. Effects of MnFe_2_O_4_/SEP on Soil pH, CEC, and Available Pb Content

**Figure 5:**
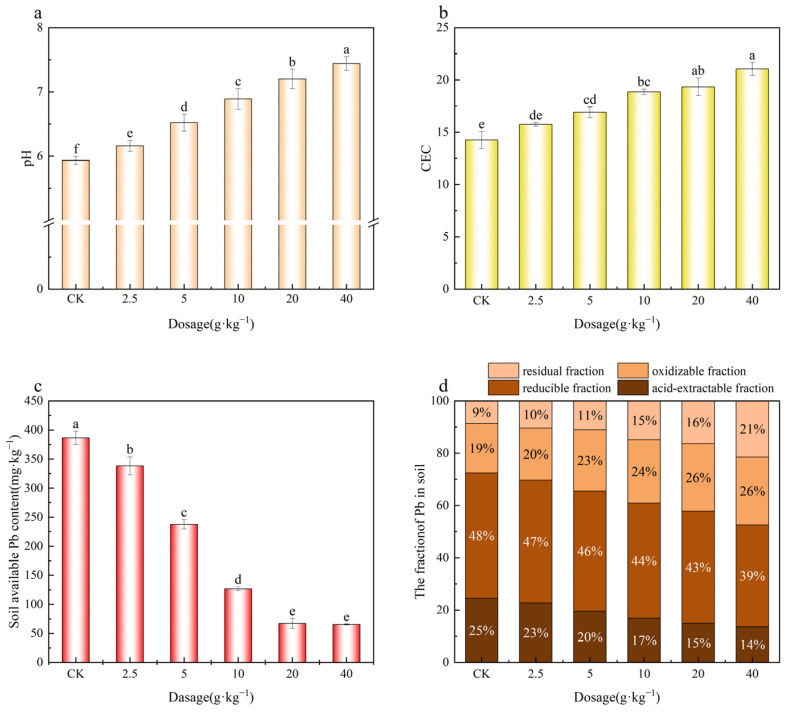
Effects of addition of different amounts of MnFe_2_O_4_/SEP on (**a**) soil pH, (**b**) soil CEC, (**c**) available Pb content, and (**d**) soil Pb fraction. Data are presented as mean ± SE (*n* = 3). Different lowercase letters indicate significant difference between different treatments (*p* < 0.05).

As shown in Figure 5, MnFe_2_O_4_/SEP increased soil pH and cation exchange capacity (CEC) in a dose-dependent manner. Compared with the control (CK), soil pH rose by 0.2–1.5 units with increasing MnFe_2_O_4_/SEP, reaching 7.4 at 40 g·kg^−1^ (*p* < 0.05). This was because SEP is weakly alkaline, and as the amount of SEP added increased, it resulted in a significant increase in soil pH [27]. Soil CEC also increased by 18–47%, likely due to the material’s high surface area [28]. Correspondingly, the content of available Pb decreased by 12–83% with increasing MnFe_2_O_4_/SEP. This reduction can be attributed to adsorption and ion exchange on MnFe_2_O_4_/SEP. Sequential extraction showed that MnFe_2_O_4_/SEP decreased acid-extractable and reducible Pb fractions while increasing oxidizable and residual fractions, indicating a transformation of Pb^2+^ into more stable, less bioavailable forms and thus reducing its uptake by plants [9].

#### 2.7.2. Effect of MnFe_2_O_4_/SEP on Pb^2+^ Uptake, Chlorophyll Content and Yield in *Brassica chinensis*

**Figure 6:**
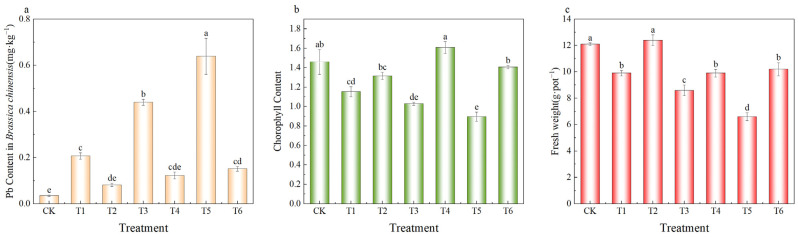
Effect of MnFe_2_O_4_/SEP on (**a**) Pb^2+^ uptake, (**b**) chlorophyll content in *Brassica chinensis* and (**c**) fresh weight of *Brassica chinensis.* Data are presented as means ± SE (*n* = 3). Different lowercase letters indicate significant difference between different treatments (*p* < 0.05). The treatments applied include: CK-control, T1—300 mg·kg^−1^ Pb^2+^, T2—300 mg·kg^−1^ Pb^2+^ plus 20 g·kg^−1^ MnFe_2_O_4_/SEP, T3—600 mg·kg^−1^ Pb^2+^, T4—600 mg·kg^−1^ Pb^2+^ plus 20 g·kg^−1^ MnFe_2_O_4_/SEP, T5—900 mg·kg^−1^ Pb^2+^, and T6—900 mg·kg^−1^ Pb^2+^ plus 20 g·kg^−1^ MnFe_2_O_4_/SEP.

As shown in Figure 6a, exogenous Pb addition (300, 600, and 900 mg·kg^−1^) significantly increased Pb uptake by *Brassica chinensis* compared with the control (CK), with Pb contents rising by 0.2, 0.4, and 0.6 mg·kg^−1^, respectively (*p* < 0.05). In contrast, MnFe_2_O_4_/SEP application reduced Pb uptake by 61%, 72%, and 76% under the corresponding Pb levels (*p* < 0.05). Pb stress also decreased chlorophyll content by 21%, 29%, and 36% (Figure 6b), while MnFe_2_O_4_/SEP increased it by 14%, 36%, and 36%, respectively, compared with treatments without the amendment. Compared with CK, Pb addition in soil significantly reduced the fresh weight of Brassica chinensis (*p* < 0.05). However, MnFe_2_O_4_/SEP treatment led to a significant increase of 15–54% in fresh weight compared with the non-MnFe_2_O_4_/SEP group (*p* < 0.05) (Figure 6c). These results indicate that MnFe_2_O_4_/SEP effectively lowers bioavailable Pb in soil, mitigates Pb-induced cellular damage, and enhances chlorophyll levels in *Brassica chinensis* [29].

#### 2.7.3. Effect of MnFe_2_O_4_/SEP on MDA and Antioxidant Enzymes System in *Brassica chinensis*

**Figure 7:**
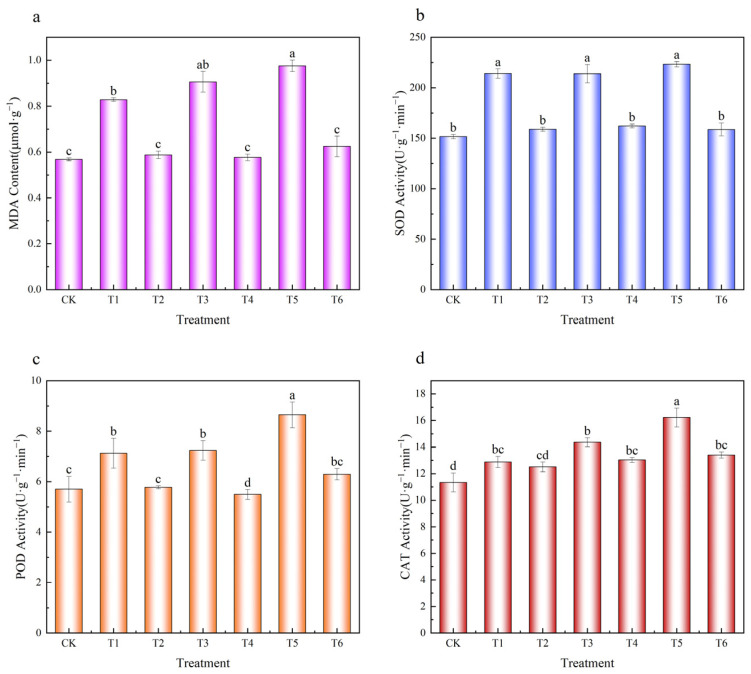
Effect of MnFe_2_O_4_/SEP on the activity of antioxidant enzymes induced in response to Pb^2+^ taken up from soil. (**a**) MDA content of *Brassica chinensis*, (**b**) SOD activity of *Brassica chinensis*, (**c**) POD activity of *Brassica chinensis*, (**d**) CAT activity of *Brassica chinensis*. Data are presented as mean ± SE (*n* = 3). Different lowercase letters indicate statistically significant differences between different treatments (*p* < 0.05). The treatments applied include: CK- control, T1—300 mg·kg^−1^ Pb^2+^, T2—300 mg·kg^−1^ Pb^2+^ plus 20 g·kg^−1^ MnFe_2_O_4_/SEP, T3—600 mg·kg^−1^ Pb^2+^, T4—600 mg·kg^−1^ Pb^2+^ plus 20 g·kg^−1^ MnFe_2_O_4_/SEP, T5—900 mg·kg^−1^ Pb^2+^, and T6—900 mg·kg^−1^ Pb^2+^ plus 20 g·kg^−1^ MnFe_2_O_4_/SEP.

As shown in Figure 7, Pb stress significantly increased MDA content in *Brassica chinensis* by 46–71% compared with the control (CK), indicating elevated lipid peroxidation. Application of MnFe_2_O_4_/SEP reduced MDA levels by 29–36%, approaching CK levels. Similarly, Pb exposure elevated the activities of antioxidant enzymes SOD, POD, and CAT, while MnFe_2_O_4_/SEP treatment effectively decreased these activities toward control levels (SOD: 24–29%; POD: 19–38%; CAT: 3–17%).

Under Pb stress, plants are compelled to generate reactive oxygen species (ROS) such as O^2−^, OH^−^, NO^−^, which disrupt the antioxidant defense system. Plants can activate their intrinsic defense mechanisms to scavenge these products and mitigate oxidative damage by enhancing the activities of key antioxidant enzymes, including SOD, CAT, and POD [30,31]. When plants are under environmental stress, MDA is produced through lipid peroxidation in cell membranes and cytoplasm. Higher MDA content indicates more severe stress damage in plants. In this study, MnFe_2_O_4_/SEP application reduced the Pb content in *Brassica chinensis*, alleviated the toxic effects of free radicals and ROS to plant cells and tissues, and enhanced SOD, CAT, and POD activities, thereby mitigating Pb stress-induced damage [32].

## 3. Materials and Methods

### 3.1. Sample Preparation and Characterization

Brown soil (0–20 cm depth) was collected from Dalian Village, Sujiatun District, Shenyang, Liaoning Province. Samples were air-dried, ground, sieved through a 20-mesh sieve, and analyzed for physicochemical properties. Soil pH was measured according to ISO 10390 [33] using a glass electrode in a 1:2.5 soil-water suspension, and organic matter by the K_2_Cr_2_O_7_ external heating method [34]. Pb, Fe, and Mn contents were determined by acid digestion method [35]. Results showed pH 5.8 ± 0.1, organic matter 34.4 ± 1.5 g·kg^−1^, Pb 34.7 ± 2.1 mg·kg^−1^, Fe 4.1 ± 0.3 g·kg^−1^, and Mn 1.0 ± 0.1 g·kg^−1^. SEP was purchased from Sinopharm Chemical Reagent Co., Ltd., Shanghai, China. *Brassica chinensis* seeds were purchased from Xinhai Agricultural Development Co., Ltd., Harbin, China.

### 3.2. Synthesis of MnFe_2_O_4_/SEP and Characterization

MnFe_2_O_4_/SEP was synthesized by co-precipitation [36]. Briefly, 0.1 mol·L^−1^ MnSO_4_·H_2_O and 0.2 mol·L^−1^ FeCl_3_·6H_2_O were mixed at a molar ratio of 1:2 and stirred at 25 °C and 200 rpm for 30 min. The pH was adjusted to 10, followed by aging at 60 °C for 4 h. The resulting suspension was filtered through a 0.45 μm membrane, washed with distilled water, and dried to obtain magnetic MnFe_2_O_4_ nanoparticles. For MnFe_2_O_4_/SEP synthesis, SEP was incorporated into the precursor solution, and the same procedure was repeated. Preliminary adsorption tests with SEP: MnFe_2_O_4_ ratios of 1:2, 1:1, and 2:1 showed that the 1:1 ratio exhibited the highest performance (248 mg·g^−1^ adsorption capacity; 99% Pb^2+^ removal) and was therefore selected for subsequent experiments.

The morphologies and elemental compositions of SEP and MnFe_2_O_4_/SEP were examined by SEM equipped with EDS (ZEISS GeminiSEM 300 Carl Zeiss AG, Oberkochen, Germany). Surface area and pore size distribution were determined with a Micrometeritics ASAP 2460 (Micromeritics Instrument Corporation, Norcross, GA, USA). Mineral phases of SEP, MnFe_2_O_4_ and MnFe_2_O_4_/SEP were analyzed by XRD (Rigaku Ultima Ⅳ Rigaku Corporation, Tokyo, Japan) with a scanning range of 2θ = 5°~90° and a rate of 5 °·min^−1^. Surface functional groups of SEP and MnFe_2_O_4_/SEP were characterized by FT-IR (Thermo Scientific Nicolet iS50 Thermo Fisher Scientific Inc., Waltham, MA, USA) in the range of 400–4000 cm^−1^. The chemical composition of MnFe_2_O_4_/SEP before and after Pb adsorption were further investigated by XPS (Thermo Scientific K-Alpha Thermo Fisher Scientific Inc., Waltham, MA, USA).

### 3.3. Adsorption Experiments: SEP and MnFe_2_O_4_/SEP Efficiency in Removal of Pb^2+^

Batch experiments were performed to investigate Pb^2+^ removal by SEP and MnFe_2_O_4_/SEP. The effects of pH (2–6), adsorbent dosage (0.2–1.0 g·L^−1^), temperature (298–318 K), and initial Pb^2+^ concentration (100–1000 mg·L^−1^) were studied in 150 mL conical flasks at 180 rpm. Simulated Pb^2+^ solutions were prepared from analytical-grade Pb (NO_3_)_2_ in deionized water. For adsorption kinetics, 0.04 g of absorbent was added to 100 mL Pb^2+^ solutions of 500 mg·L^−1^, and samples were collected at various time points within 24 h. After filtration through 0.45 μm syringe filters, Pb^2+^ concentrations were determined by atomic absorption spectrophotometry (Agilent240FS Agilent Technologies, Inc., Santa Clara, CA, USA). All experiments were performed in triplicate. Adsorption capacity calculations are detailed in the Supplemental Information (Text S1).

### 3.4. Regeneration and Reuse Efficiency of MnFe_2_O_4_/SEP

Regeneration and reuse were evaluated through repeated adsorption–desorption cycles. For adsorption, 0.5 g of MnFe_2_O_4_/SEP was dispersed in 100 mL Pb^2+^ solution (500 mg·L^−1^) and stirred for 24 h. The mixture was filtered through a 0.45 μm syringe filter, and the adsorbent was dried at 105 °C for 24 h. Pb^2+^ concentrations before and after adsorption were measured by flame atomic absorption spectrometry (Agilent 240FS) to calculate removal rate efficiency. For desorption, the dried adsorbent was treated to 100 mL of 0.2 mol·L^−1^ EDTA-2Na solution for 24 h, filtered, and dried again. Pb^2+^ concentration in the desorption solution was measured, and the recovery rate was calculated as the ratio of desorbed Pb^2+^ to previously adsorbed Pb^2+^. The adsorption–desorption cycle was repeated five times. Calculation details are provided in the Supplemental Information (Text S1).

### 3.5. Soil Incubation Experiment

Pb-contaminated soil was prepared by spiking with a Pb (NO_3_)_2_ to achieve 600 mg·kg^−1^, simulating moderately contaminated farmland soil according to the Soil Environmental Quality Risk Control Standard for Agricultural Land (GB 15618-2018) [37], intervention range 400–1000 mg·kg^−1^). The spiked soil was air-dried for 15 days and passed through a 20-mesh sieve. For incubation, 100 g of soil was thoroughly mixed with MnFe_2_O_4_/SEP at 0, 2.5, 5, 10, 20, and 40 g·kg^−1^and transferred into 300 mL plastic bottles, with three replicates per treatment. Soil moisture was maintained at 60% of water holding capacity by periodic addition of deionized water over 30 days.

After incubation, the soil pH, CEC, available Pb, Pb fractionation, and total Pb were determined. CEC was measured by the BaCl_2_-H_2_SO_4_ compulsive exchange method (ISO 11260, 2017) [9]. Available Pb was extracted using DTPA according to the Chinese National Standard GB/T 23739-2009 [38], Pb fractionation was determined via the European Community Bureau of Reference (BCR) sequential extraction procedure [39], and total Pb was measured by strong acid digestion (GB/T 17141-1997) [35].

### 3.6. Pot Experiments for Remediation Assessment

Simulated Pb-contaminated soils were prepared by spiking with Pb(NO_3_)_2_ to final concentrations of 300, 600, and 900 mg·kg^−1^. Treatments included soils with or without 20 g·kg^−1^ MnFe_2_O_4_/SEP, with three replicates per treatment. The experimental design consisted of the following treatment: 300 mg·kg^−1^ Pb without MnFe_2_O_4_/SEP (T1), 300 mg·kg^−1^ Pb with 20 g·kg^−1^ MnFe_2_O_4_/SEP (T2), 600 mg·kg^−1^ Pb without MnFe_2_O_4_/SEP (T3), 600 mg·kg^−1^ Pb with 20 g·kg^−1^ MnFe_2_O_4_/SEP (T4), 900 mg·kg^−1^ Pb without MnFe_2_O_4_/SEP (T5), and 900 mg·kg^−1^ Pb with 20 g·kg^−1^ MnFe_2_O_4_/SEP (T6). A control (CK) without Pb^2+^ or MnFe_2_O_4_/SEP was also included. *Brassica chinensis* was selected as the test plant. A uniform amount of compound fertilizer was applied to each pot. After thorough mixing, the soils were equilibrated for one week before sowing. Ten seeds were sown per pot, and seedlings were thinned to five uniform plants after emergence. Soil moisture was maintained at 60% of the water holding capacity by watering every two days. After 60 days of growth, the entire plants were harvested, and fresh weights were measured using an electronic balance. Samples that were not analyzed immediately were temporarily stored at 4 °C for subsequent determination of Pb accumulation, chlorophyll content, MDA concentration, and antioxidant enzyme activities.

For Pb determination, the leaves were oven-dried at 105 °C for 30 min, followed by drying at 65 °C to a constant weight before further processing. The dried samples were then ground into a fine powder and digested with 2 mL of HClO_4_ and 8 mL of HNO_3_ for 12 h. Solutions were filtered through a 0.45 μm syringe filter prior to Pb determination by ICP-MS (Agilent 7500 Agilent Technologies, Inc., Santa Clara, CA, USA) [40]. Chlorophyll content was measured using the spectrophotometric method [9]. The contents of MDA, SOD, POD, and CAT activities were determined using the trichloroacetic acid-thiobarbituric acid (TCA-TBA), nitroblue tetrazolium (NBT) photoreduction, guaiacol, and hydrogen peroxide methods, respectively [9].

### 3.7. Statistics and Analysis

Isothermal adsorption data were fitted using the Langmuir, D-R model, and Temkin model and adsorption kinetics were analyzed using pseudo-first-order and pseudo-second-order models. Thermodynamic parameters were calculated using the Van’t Hoff equation. Detailed equations and models are provided in Supplemental Information (Text S2). Data processing was performed with Microsoft Excel 2019 and SPSS 26. One-way ANOVA followed by Duncan’s multiple range test (*p* = 0.05) was applied to assess significant differences between treatments. Figures were compiled using Origin 2022.

## 4. Conclusions

These results demonstrated that a SEP-supported MnFe_2_O_4_ composite was effectively synthesized by chemical co-precipitation using SEP as the raw material, as confirmed by SEM-EDS, XRD, FT-IR and XPS analyses. The MnFe_2_O_4_/SEP composite showed enhanced Pb^2+^ adsorption performance compared with pure SEP under various experimental conditions. Thermodynamic analysis indicated that the adsorption process was spontaneous and primarily governed by chemisorption through ion exchange between Pb^2+^ and H^+^, with physisorption playing a secondary role. Moreover, the composite demonstrated excellent reusability, maintaining a high Pb^2+^ removal efficiency (85.4%) after five consecutive adsorption–desorption cycles. In Pb-contaminated soils, application of 20 g·kg^−1^ MnFe_2_O_4_/SEP effectively improved soil pH and CEC, reduced Pb bioavailability, and alleviated Pb-induced oxidative stress in *Brassica chinensis*.

Furthermore, due to its high surface area, abundant functional groups, and strong cation-exchange capacity, MnFe_2_O_4_/SEP has the potential to immobilize other heavy metals, such as Cd^2+^ and Cu^2+^, although further studies are required to confirm its effectiveness and selectivity. Future research should focus on large-scale field trials, long-term stability under variable environmental conditions, and the assessment of potential ecological impacts. Limitations of this study include the controlled laboratory conditions, short-term exposure, and evaluation of only one crop species, which may not fully reflect complex field scenarios. Addressing these aspects will be crucial for practical application in contaminated soils.

## Figures and Tables

**Table 1:** Langmuir, Dubinin-Radushkevich and Temkin parameters for Pb^2+^ adsorption onto MnFe_2_O_4_/SEP.

T(K)	Langmuir			D-R			Temkin		
	q_max_ (mg·g^−1^)	K_L_ (L·mg^−1^)	R^2^	q_max_ (mg·g^−1^)	E (kJ·mol^−1^)	R^2^	A (L·mg^−1^)	b (kJ·mol^−1^)	R^2^
298	458.72	0.09	0.99	368.5	0.37	0.88	2.26	28.01	0.91
308	500.00	0.08	0.99	380.0	0.43	0.86	1.94	30.06	0.95
318	549.45	0.07	0.99	413.5	0.49	0.87	1.83	31.80	0.97

## Data Availability

Data are contained within the article.

## Supplementary Materials

The following supporting information can be downloaded at: https://www.mdpi.com/article/10.3390/plants14193077/s1, Calculation of the adsorption capacity of the adsorbents, the Pb^2+^ removal rate, and the Pb^2+^ recovery rate is provided in Text S1. Kinetics and thermodynamics analysis models and function equations is provided in Text S2. The structural characteristics of the SEP and MnFe_2_O_4_/SEP materials are provided in Table S1. The content of each element in SEP and MnFe_2_O_4_/SEP is provided in Table S2. Pseudo-first-order dynamics and pseudo-second-order kinetics parameters for Pb^2+^ adsorption on MnFe_2_O_4_/SEP is provided in Table S3. Comparison of the physico-chemcial characteristics of SEP and MnFe_2_O_4_/SEP. (a, b) SEM images of SEP and MnFe_2_O_4_/SEP; (c, d) EDS images of SEP and MnFe_2_O_4_/SEP; (e) XRD patterns of SEP, MnFe_2_O_4_, and MnFe_2_O_4_/SEP, and (f) FT-IR spectra of SEP and MnFe_2_O_4_/SEP is provided in Figure S1. Linear fitting of the adsorption process to (a) pseudo-first-order and (b) pseudo-second-order kinetic models is provided in Figure S2.
