# Supplementary material for: Lead Immobilization in Soil and Uptake Reduction in Brassica chinensis Using Sepiolite-Supported Manganese Ferrite
Source: Plants (Basel). 2025 Oct 5;14(19):3077. doi: 10.3390/plants14193077 (PMC12526466; doi:10.3390/plants14193077)
Supplement: Supplementary file 1 [file plants-14-03077-s001.zip › plants-3865747-supplementary.pdf]

Lead Immobilization in Soil and Uptake Reduction in *Brassica chinensis* Using Sepiolite-Supported Manganese Ferrite

Fengzhuo Geng[1, †], Yaping Lyu[2, †], Liansheng Ma[1], Yin Zhou[1], Jiayue Shi[1], Roland Bol[3], Peng Zhang[2], Iseult Lynch[4], Xiuli Dang[1]*

[1] National Engineering Research Center for Efficient Utilization of Soil and Fertilizer Resources, Key Laboratory of Arable Land Conservation in Northeast China, Ministry of Agriculture and Rural Affairs, College of Land and Environment, Shenyang Agricultural University, Shenyang 110866, P. R. China

[2] Department of Environmental Science and Engineering, University of Science and Technology of China, Hefei 230026, P. R. China

[3] Institute of Bio- and Geosciences, Agrosphere (IBG-3), Forschungszentrum Jülich GmbH, Jülich 52425, Germany

[4] School of Geography, Earth and Environmental Sciences, University of Birmingham, Edgbaston, Birmingham, B15 2TT, UK

Correspondence: dxl@syau.edu.cn；Tel.:+86-136-0492-2085

[†] The authors contributed equally to this paper *

The number of pages: 5

The number of text: 2

The number of table: 3

The number of figures:2

List of Supporting Information

Text:

Text S1 Calculation of the adsorption capacity of the adsorbents, the Pb^{2+} removal rate, and the Pb^{2+} recovery rate

Text S2 Kinetics and thermodynamics analysis models and function equations

Table:

Table S1 The structural characteristics of the SEP and $MnFe_2O_4$/SEP materials.
Table S2 The content of each element in SEP and $MnFe_2O_4$/SEP.
Table S3 Pseudo-first-order dynamics and pseudo-second-order kinetics parameters for Pb^{2+} adsorption on $MnFe_2O_4$/SEP.

Figures:

Figure. S1. Comparison of the physico-chemcial characteristics of SEP and $MnFe_2O_4$/SEP. (a, b) SEM images of SEP and $MnFe_2O_4$/SEP; (c, d) EDS images of SEP and $MnFe_2O_4$/SEP; (e) XRD patterns of SEP, $MnFe_2O_4$, and $MnFe_2O_4$/ SEP, and (f) FT-IR spectra of SEP and $MnFe_2O_4$/SEP.
Figure. S2 Linear fitting of pseudo-first-order (a) and pseudo-second-order kinetic models (b) in the adsorption process of Pb^{2+} onto $MnFe_2O_4$/SEP.

Text S1 The calculation of the adsorption capacity of the adsorbents and the Pb^{2+} removal rate.

The equation of adsorption capacity of adsorbents:

$$q = \frac{(C_0 - C_t) \times V}{m \times 1000} \quad (S1)$$

The equation of removal rate of Pb^{2+}:

$$r_1 = \frac{C_0 - C_t}{C_0} \times 100\% \quad (S2)$$

The equation of recovery rate of Pb^{2+}:

$$r_2 = \frac{C_0 - C_t}{C_{des}} \times 100\% \quad (S3)$$

where q is the adsorption quantity ($mg \cdot g^{-1}$); c_0 is the initial Pb^{2+} concentration ($mg \cdot L^{-1}$); c_t is the final Pb concentration after adsorption ($mg \cdot L^{-1}$); V is the volume of the Pb solution; m is the dosage of materials (g); r_1 is the removal rate; r_2 is the recovery rate; c_{des} is the Pb^{2+} concentration in the desorption solution.

Text S2 Analysis model and function equation.

The isothermal adsorption model of Pb^{2+} by $MnFe_2O_4$/SEP was fitted by the Langmuir, Dubinin-Radushkevich and Temkin models.

The linear expression of Langmuir's model is as follows:

$$\frac{c_e}{q_e} = \frac{1}{K_L q_{max}} + \frac{c_e}{q_{max}} \quad (S4)$$

where c_e is the Pb concentration at equilibrium ($mg \cdot L^{-1}$); q_e is the adsorption quantity at equilibrium ($mg \cdot g^{-1}$); q_{max} is the maximum adsorption capacity ($mg \cdot g^{-1}$) and K_L is the Langmuir constant ($L \cdot g^{-1}$), which is related to the binding energy of the metal ions to the active site.

The linear expression of the Dubinin-Radushkevich model is as follows:

$$lnq_e = lnq_{max} - \beta \varepsilon^2 \quad (S5)$$

$$\varepsilon = RTln(1 + \frac{1}{c_e}) \quad (S6)$$

$$E = \frac{1}{\sqrt{2\beta}} \quad (S7)$$

where q_e is the adsorption quantity at equilibrium ($mg \cdot g^{-1}$); q_{max} is the maximum adsorption capacity ($mg \cdot g^{-1}$); β is the adsorption performance constant ($mol^2 \cdot kJ^{-2}$); ε is the Polanyi adsorption potential ($kJ \cdot mol^{-1}$); R is the ideal gas constant (8.314 $J \cdot mol^{-1} \cdot K^{-1}$); T is the absolute temperature (K); c_e is the equilibrium concentration ($mol \cdot L^{-1}$); E is the average adsorption free energy ($kJ \cdot mol^{-1}$).

The linear expression of the Temkin model is as follows:

$$q_e = BlnA + Blnc_e \quad (S8)$$

$$B = \frac{RT}{b} \quad (S9)$$

where q_e is the adsorption quantity at equilibrium (mg·g^{-1}); c_e is the equilibrium concentration (mol·L^{-1}); A is the Temkin equilibrium binding constant (L·mol^{-1}); B is the constant related to adsorption heat (mg·g^{-1}); R is the ideal gas constant (8.314 J·mol^{-1}·K^{-1}); b is the Temkin adsorption heat constant (J·mol^{-1}).

The adsorption kinetics of Pb^{2+} by $MnFe_2O_4$/SEP were fitted by a pseudo-first-order kinetic equation and a pseudo-second-order kinetic equation.

Pseudo-first-order kinetic equation:

$$\ln(q_e - q_t) = \ln q_e - k_1 t \quad (S10)$$

Pseudo-second-order kinetic equation:

$$\frac{t}{q_t} = \frac{1}{k_2 q_e^2} + \frac{t}{q_e} \quad (S11)$$

where k_1 is pseudo-first-order reaction rate constant (min^{-1}); k_2 is pseudo-first-order reaction rate constant (mg·g^{-1}·min^{-1}).

The thermodynamic process of adsorption adopts the Van't Hoff equation.

Van't Hoff equation:

$$\ln K_d = \frac{\Delta S}{R} - \frac{\Delta H}{RT} \quad (S12)$$

$$\Delta G = \Delta H - T\Delta S \quad (S13)$$

where R (8.3145 J·mol^{-1}·K^{-1}) is the universal gas constant, T is the temperature in Kelvin, and K_d is the adsorption equilibrium constant.

Table S1 The structural characteristics of the SEP and $MnFe_2O_4$/SEP materials

Adsorbent	Surface area (m^2·g^{-1})	Pore volume (cm^3·g^{-1})	Average pore diameter (nm)
SEP	34.04±1.49	0.33±0.01	11.26±0.46
$MnFe_2O_4$/SEP	112.61±0.59	0.21±0.02	7.41±0.35

Table S2 The content of each element in SEP and $MnFe_2O_4$/SEP

Material	Element	Series	Wt%
SEP	O	K-series	42
	Mg	K-series	17
	Si	K-series	40
$MnFe_2O_4$/SEP	O	K-series	36
	Mg	K-series	11
	Si	K-series	19
	Mn	K-series	11
	Fe	K-series	22

Table S3 Pseudo-first-order dynamics and pseudo-second-order kinetics parameters for Pb^{2+} adsorption on $MnFe_2O_4$/SEP.

C_0 (mg/L)	Pseudo-first order			Pseudo-second order		
	k_1 (min^{-1})	qe ($mg\cdot g^{-1}$)	R^2	k_2 ($g\cdot mg^{-1}\cdot min^{-1}$)	qe ($mg\cdot g^{-1}$)	R^2
500	0.02	106.98	0.97	0.0009	306.75	0.99

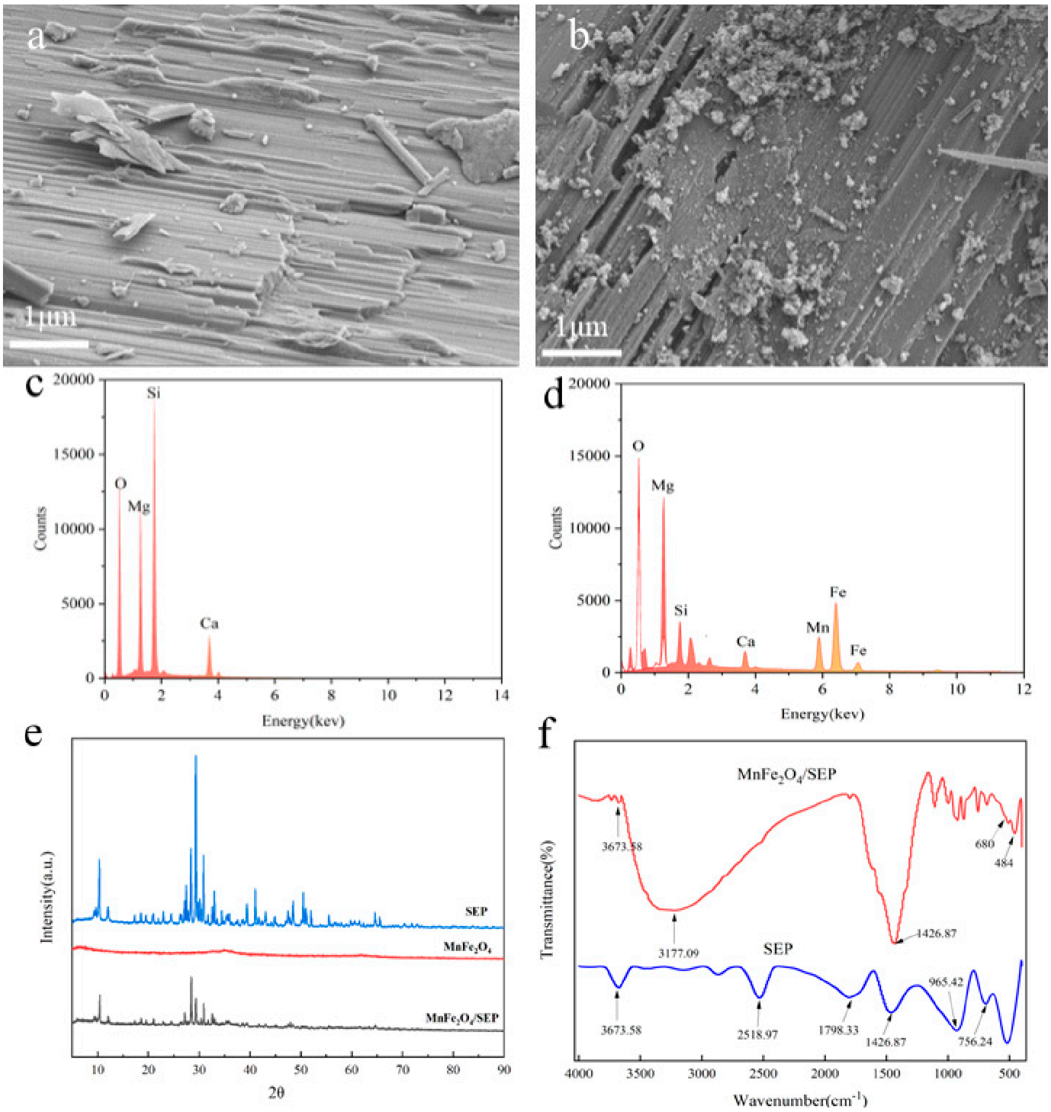

Figure. S1. Comparison of the physico-chemcial characteristics of SEP and $MnFe_2O_4$/SEP. (a, b) SEM images of SEP and $MnFe_2O_4$/SEP; (c, d) EDS images of SEP and $MnFe_2O_4$/SEP; (e) XRD patterns of SEP, $MnFe_2O_4$, and $MnFe_2O_4$/ SEP, and (f) FT-IR spectra of SEP and $MnFe_2O_4$/SEP.

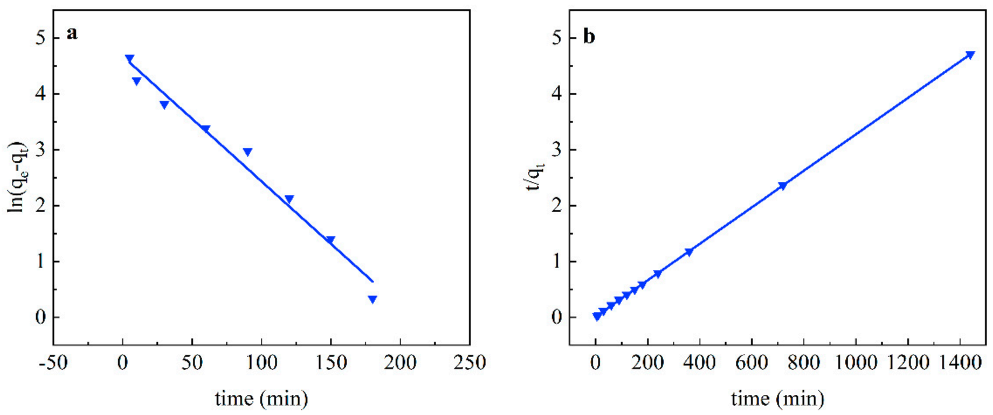

Figure. S2. Linear fitting of pseudo-first-order (a) and pseudo-second-order kinetic models (b) in the adsorption process of Pb^{2+} onto $MnFe_2O_4$/SEP.
